# Mechanisms of Granulosa Cell Programmed Cell Death and Follicular Atresia in Polycystic Ovary Syndrome

**DOI:** 10.33549/physiolres.935485

**Published:** 2025-02-01

**Authors:** Yu-Han SHEN, Sui PENG, Ting ZHU, Ming-Jie SHEN

**Affiliations:** 1 Department of Gynecology, Shuguang Hospital Affiliated to Shanghai University of Traditional Chinese Medicine, Shanghai, China

**Keywords:** Follicular atresia, Hyperandrogenism, Insulin resistance, Polycystic ovary syndrome, Programmed cell death of granulosa cells

## Abstract

Polycystic ovary syndrome (PCOS) is a common endocrine disorder affecting women of reproductive age, characterized by a spectrum of reproductive, endocrine, and metabolic disturbances. The etiology of PCOS encompasses a complex interplay of genetic, metabolic, inflammatory, and oxidative factors, though the precise pathological mechanisms remain inadequately understood. Despite considerable variability in the clinical characteristics and biochemical profiles among individuals with PCOS, abnormalities in follicular development are a hallmark of the condition. Granulosa cells, integral to follicular development, play a pivotal role in follicle maturation. Recent studies have established a strong correlation between granulosa cell programmed cell death and follicular atresia in PCOS. This review provides a comprehensive analysis of the current understanding of granulosa cell programmed cell death and its contribution to follicular atresia within the pathophysiology of PCOS, providing a foundation for future research endeavors.

## Introduction

Polycystic ovary syndrome (PCOS) is a prevalent endocrine and reproductive disorder affecting women of reproductive age globally [1–3]. With a prevalence ranging from 5 % to 10 % among women of childbearing age, PCOS is characterized by clinical or biochemical manifestations of androgen excess, oligo-ovulation or anovulation, and polycystic ovarian morphology. It is the leading cause of ovulatory infertility [1–4]. While the clinical and biochemical manifestations of PCOS are highly heterogeneous, disturbances in follicular development, particularly during antral follicle selection, are core features of this primary ovarian disorder [5–7]. As critical components of the follicular microenvironment granulosa cells facilitate bidirectional communication with oocytes through gap junctions, thereby promoting follicular maturation and oocyte maturation [8]. For instance, granulosa cells secrete nutrients and molecular signals, including sex steroids, growth factors, cytokines, bioactive peptides, and proteins. These factors are crucial for regulating follicular initiation, recruitment growth, and development through gap junctional communication [9]. Moreover, granulosa cells secrete paracrine growth factors, undergo self-renewal, and influence oocyte growth and maturation. For instance, they secrete factors that initiate primordial follicle activation and regulate oocytes quiescence or awakening through mTORC1-mediated interactions between C-Kit proto-oncogene protein ligand (KITL) and C-Kit proto-oncogene protein (KIT) [10].

However, follicular development disorders, particularly follicular atresia, are primary factors contributing to reduced fertility in women with PCOS. The underlying mechanisms are complex and involve multiple regulatory factors closely associated with granulosa cell programmed cell death. Granulosa cell programmed cell death encompasses apoptosis, autophagy, ferroptosis, necroptosis, and pyroptosis. Various factors, including hormonal imbalances, cytokines, oxidative stress, chronic inflammation, and mitochondrial damage, have been implicated in inducing granulosa cell programmed cell death through the regulation of intrinsic cellular pathways, thereby contributing to the process of follicular atresia in PCOS [11–14]. Consequently, it is essential to have a comprehensive investigation of the mechanisms underlying programmed cell death and follicular atresia in granulosa cells of patients with PCOS.

## Excessive granulosa cell apoptosis and follicular atresia in PCOS

Apoptosis is a regulated, active form of programmed cell death. Characteristic morphological changes including cell contraction, chromatin condensation (pyknosis), membrane blebbing, and mitochondrial swelling occur during apoptosis. These changes facilitate efficient clearance of apoptotic cells, maintaining internal homeostasis [15]. However, excessive apoptosis of granulosa cells can result in follicular atresia. Studies indicate that 10 % apoptotic rate within a follicle initiates an atretic process [11,14]. The primary apoptotic pathways involved are the mitochondria-mediated intrinsic pathway and the death receptor-mediated extrinsic pathway, both culminating in caspase activation [11]. Consequently, follicular atresia arises from granulosa cell apoptosis induced by a complex interplay of endogenous and exogenous factors, activating intricate regulatory networks and molecular mechanisms.

Excessive granulosa cell apoptosis in PCOS follicles is closely related to the following mechanisms:

### Hyperandrogenism induced excessive apoptosis of granular cells

Androgens include testosterone, androstenedione, dehydroepiandrosterone (DHEA), and dehydroepiandrosterone sulfate (DHEAS). Hyperandrogenism is one of the key characteristics of PCOS. The primary sources of androgens in women are the ovaries and adrenal glands. Elevated anti-Müllerian hormone (AMH) levels in PCOS patients lead to disrupted follicular development, with excessive synthesis of androgens by theca cells in the ovaries, which in turn induces granulosa cells to secrete more AMH. On the other hand, hyperandrogenemia decreases the sensitivity of the hypothalamus to estradiol and progesterone, resulting in increased levels of gonadotropin-releasing hormone (GnRH) and luteinizing hormone (LH). This acceleration in the production of preantral and small antral follicles leads to follicular developmental disorders [16]. But recently studies have linked follicular development abnormalities in patients with PCOS to hyperandrogenism-induced excessive granulosa cell apoptosis. Hyperandrogenism upregulates dynamin-related protein 1 (Drp1) expression in granulosa cells of PCOS rats, a protein associated with mitochondrial fission. This alteration disrupts the equilibrium between mitochondrial fusion and fission, resulting in excessive mitochondrial fragmentation. This morphological change promotes the release of cytochrome c (Cyt c), triggering a cascade and culminating in granulosa cell apoptosis [17]. Secondly, hyperandrogenism-induced alterations in mitochondrial membrane potential, contributing to granulosa cell apoptosis, are associated with abnormal heat shock protein (HSPs) expression within follicles. HSP10, a critical HSP family member, exhibits cytoprotective functions under stress conditions such as UV exposure and hypoxia [18]. Ni *et al*. reported a significantly decreased HSP10 expression in granulosa cells of PCOS mice compared to normal controls, resulting in diminished cytoprotective effects [19]. Concurrently, increased Bax expression, decreased Bcl-2 levels, and an elevated Bax/Bcl-2 ratio were observed. These changes disrupted mitochondrial transmembrane potential, leading to Cyt c release into the cytoplasm. Consequently, activation of caspase-3 and caspase-9 led to inducing apoptosis in granulosa cells.

### Oxidative stress promotes excessive granulosa cell apoptosis

Oxidative stress is characterized by an imbalance between reactive oxygen species (ROS) production and antioxidant capacity, and contributes to tissue and cellular damage [20]. In the context of PCOS, suppressed glycolytic function in granulosa cells and elevated follicular fatty acids exacerbate the oxidative stress [21,22]. The resulting ROS-induced mitochondrial dysfunction characterized by mitochondrial membrane, increases the permeability of mitochondrial membranes. This leads to Cyt c release and subsequent activation of the caspase cascade, ultimately promoting granulosa cell apoptosis.

### Chronic inflammation promotes granulosa cell apoptosis

Patients with PCOS often exhibit a chronic inflammatory state attributed to factors such as obesity, elevated androgen levels, and insulin resistance [23]. Chronic inflammation is also an important factor contributing to granulosa cell atresia. A study has found that mouse models treated with testosterone exhibit overexpression of serum interleukin-6 (IL-6), IL-1β, and nucleotide-binding oligomerization domain-like receptor protein 3 (NLRP3) inflammasomes. Overexpression of NLRP3 significantly enhances the expression of fibrotic factors such as transforming growth factor β (TGF-β) and connective tissue growth factor (CTGF) in ovarian cells. When NLRP3 expression is inhibited, dihydrotestosterone (DHT)-treated granulosa cells show significant reductions in NLRP3, TGF-β, and CTGF at the protein level. This demonstrates that hyperandrogenism stimulates chronic low-grade inflammation in the ovary to activate NLRP3, further inducing apoptosis of ovarian granulosa cells and ovarian fibrosis [24].

Furthermore, another study has found that the inflammatory milieu inhibits granulosa cell proliferation. Pro-inflammatory cytokines induce mitochondrial apoptosis by downregulating Bcl-2 mRNA expression and upregulating Bax mRNA expression. The subsequent activation of the caspase family culminates in granulosa cells apoptosis [25].

### Effects of environmental endocrine disruptors on granulosa cell apoptosis

Endocrine disrupting chemicals (EDCs) are a class of chemicals ubiquitous in both human living environments and nature. They can interfere with the function of hormone receptors, affect hormone synthesis and metabolism, and regulate the function of hormone-sensitive cells and tissues [26]. Currently, EDCs associated with PCOS include phenolic compounds (such as bisphenol A (BPA), bisphenol S (BPS), bisphenol Z (BPZ), and bisphenol AF (BPAF)) [27–30], phthalates and their metabolites (like di(2-ethylhexyl) phthalate (DEHP) and mono-methyl-phthalate (MMP)) [31,32], perfluoroalkanes (such as 6:2 chlorinated perfluoroalkyl ether sulfonic acid (6:2 Cl-PFESA) and hexafluoropropylene oxide dimer acid (HFPO-DA)) [33], and other organic pollutants (such as the organic UV filter octocrylene [34], p, p'-dichlorodiphenyltrichlo-roethane (p, p'-DDT), o, p'-DDT, and p, p'-dichlorodiphe-nyldichloroethylene (p, p'-DDE) [35]. A recent study has found that rats exposed to BPA during adulthood can exhibit increased serum luteinizing hormone (LH) levels and altered gene expression levels of various steroid hormone synthase enzymes in the ovaries, leading to disruption of the estrous cycle, ovarian atrophy, and an increase in the number of follicles and cystic follicles [36]. Propylparaben (PrPB) can cause increased oxidative damage to proteins and lipids in the ovaries and also induce structural changes in mitochondria and reduced ATP production in ovarian granulosa cells of mice [37]. DEHP not only inhibits granulosa cell proliferation by upregulating the expression of four microRNAs [38] but also activates the NF-κB signaling pathway in mouse ovaries, upregulates the expression of the NLRP3 inflammasome, and promotes granulosa cell pyroptosis [39]. BPA can promote autophagy in mouse ovarian granulosa cells by activating the AMPK/mTOR/ULK1 signaling pathway [40]. These EDCs all contribute to the pathogenesis of PCOS by promoting ovarian granulosa cell apoptosis, causing follicular atresia, and participating in the development of PCOS.

### The impact of Anti-Müllerian hormone (AMH) on granulosa cell apoptosis

AMH is a glycoprotein primarily secreted by granulosa cells in preantral and small antral follicles of the ovary. PCOS is characterized by early follicular developmental disorders, which are associated with elevated AMH levels [41]. A study has found that high levels of AMH can lead to abnormal activation of granulosa cell autophagy by activating the autophagy-related gene BECN1 mRNA. This, in turn, causes apoptosis of preantral follicle granulosa cells, resulting in follicular atresia and preventing the transition of antral follicles into preovulatory follicles, thereby further exacerbating follicular developmental disorders in PCOS [42]. As previously discussed, high levels of AMH can also cause excessive synthesis of androgens by theca cells, further leading to granulosa cell apoptosis and follicular atresia.

### Effects of vitamin D on granulosa cell proliferation

A recent research has revealed that vitamin D promotes follicular development and ovulation, directly increasing the thickness of the granulosa layer of follicles [43]. Its mechanism of action may be closely related to vitamin D's ability to enhance the expression of microRNA-196-5p, inhibit apoptosis in human ovarian granulosa cells, promote proliferation, suppress the generation of reactive oxygen species (ROS), and facilitate glucose uptake, thereby promoting granulosa cell growth [44]. Additionally, vitamin D's influence on granulosa cell development may also occur by elevating the levels of sex hormone-binding globulin (SHBG), thereby improving the hyperandrogenic manifestations in patients with polycystic ovary syndrome (PCOS), further affecting granulosa cell proliferation. Studies have indicated that serum levels of 25-hydroxyvitamin D are significantly positively correlated with SHBG levels and significantly negatively correlated with free testosterone levels [45].

### Effects of obesity on granulosa cell apoptosis

Beyond causing insulin resistance and hyperandrogenemia, obesity influences granulosa cell development and function through multiple pathways. In obese patients, the expression level of fatty acid translocase CD36 in granulosa cells is higher than that in patients of normal weight. Overexpression of CD36 inhibits granulosa cell proliferation and promotes granulosa cell apoptosis [46]. Meanwhile, obese women have higher levels of oxidized low-density lipoprotein (oxLDL) in both serum and preovulatory follicles. The oxidative stress response caused by increased levels of lipid peroxides and circulating oxLDL can inhibit the differentiation of luteinized granulosa cells in mice [47]. Furthermore, human obesity is often associated with hyperleptinemia (leptin resistance). High leptin levels in follicular fluid significantly inhibit granulosa cell proliferation. The mechanism may involve high leptin levels activating the granulosa cell apoptosis process, reducing Bcl-2 expression, upregulating Bax levels, leading to an imbalance in the ratio of anti-apoptotic and pro-apoptotic proteins, activating downstream caspase-3, and inducing granulosa cell apoptosis [48].

## Excessive autophagy in granulosa cells and follicular atresia in PCOS

Autophagy is a tightly regulated cellular process involving the degradation and recycling of cytosolic components, including damaged organelles and macromolecules, within lysosomes. While basal autophagy is crucial for maintaining cellular homeostasis, its dysregulation can precipitate cell death [49]. Autophagy is a cellular process involving the lysosomal degradation and recycling of cellular components, including damaged organelles, misfolded proteins, and lipid droplets [50]. Essential for maintaining cellular homeostasis during follicular development, autophagy plays a pivotal role in granulosa cell function, contributing to primordial follicle maintenance, germ cell survival, and luteal remnants removal. However, excessive autophagy in granulosa cells can negatively impact the quality and quantity of follicular cells, resulting in ovarian tissue damage and decreased reproductive performance in animals [51].

Excessive autophagy in granulosa cells in PCOS follicles is closely related to the following mechanisms:

### Insulin resistance and excessive autophagy in granular cells

PCOS is a follicular development disorder closely linked to insulin resistance (IR). IR within ovarian granulosa cells of patients with PCOS is a primary underlying mechanism. Ni *et al*. demonstrated a positive correlation between excessive granulosa cell apoptosis and IR in PCOS patients [19]. Furthermore, IR promotes granulosa cell apoptosis through abnormal autophagic activation. Bcl-2 expression in granulosa cells is suppressed by accumulating autophagosomes [52]. Beclin-1, a key autophagy regulator, interacts with Bax, Bcl-2, and activated caspase-3 within the Bcl-2 family. This interaction induces Bax release and translocation from cytoplasm to the outer mitochondrial membrane. Consequently, this process promotes Cyt c release from mitochondria, activating the caspase family, triggering the caspase cascade, and ultimately inducing granulosa cell apoptosis [53].

### Androgen and excessive autophagy in granular cells

Hyperandrogenism, a predominant clinical manifestation of PCOS, is associated with accelerated follicular atresia. This process is closely linked to excessive autophagy within granulosa cells. Li *et al*. reported a positive correlation between Beclin1 mRNA, an autophagy marker, and serum total testosterone levels in human granulosa cells [54]. Additionally, DHT upregulated Beclin1 mRNA expression and increased the light chain 3 (LC3) type II to type I ratio in a dose-dependent manner within cultured granulosa cells inducing excessive autophagy. In DHT rat model, Salehi *et al*. observed upregulated Drp1 accelerated mitochondrial fission, excessive granulosa cell autophagy, and subsequent early antral follicle growth arrest [17]. Additionally, androgens significantly upregulate ferredoxin 1 (FDX1) in granulosa cells enhancing granulosa cell autophagy, a process implicated in PCOS pathogenesis [55].

## Ferroptosis in granulosa cells and follicular atresia in PCOS

Ferroptosis is a form of programmed cell death characterized by iron-dependent lipid peroxidation and ROS accumulation, culminating in cell membrane disruption [56]. Cells undergoing ferroptosis exhibit distinct ultrastructural mitochondrial abnormalities, including condensation, swelling, increased membrane density, reduced or absent cristae, and outer membrane rupture. A clinical study involving 149 patients with PCOS and 108 controls revealed significantly higher serum ferritin levels in the PCOS group, irrespective of obesity status [57]. Multiple studies have linked iron overload in PCOS oligomenorrhea, hyperandrogenism, and insulin resistance [58–60]. Zhang *et al*. demonstrated that transferrin receptor-mediated increased iron uptake in granulosa cells promotes ROS generation, mitophagy activation, lipid peroxidation, and ultimately ferroptosis, in human ovarian granulosa cells. This process inhibits follicular development and contributes to follicular atresia [61]. Liu *et al*. further established that ferroptosis inhibitors can ameliorate PCOS-like symptoms and protect ovarian tissue from PCOS-induced damage by modulating oxidative stress, mitochondrial membrane potential, inflammation, and apoptosis [62]. Zhang *et al*. established another PCOS rat model through co-treatment with 5α-dihydrotestosterone and insulin [63]. This study identified characteristics of ferroptosis, including decreased glutathione peroxidase 4 and glutathione (GSH) levels in uterine and placental tissues. Additionally, aberrant expression of ferroptosis-related genes such as Acsl4, Tfrc, Slc7a11, and Gclc was observed. Zhang *et al*. reported involvement of circulating RNAs in PCOS development through ferroptosis [64]. CircRHBG, upregulated in the granulosa cells of individuals with PCOS, was found to inhibit ferroptosis by competing with Slc7a11 for miR-515-5p binding.

## Necrotizing apoptosis of granulosa cells and follicular atresia

Necroptosis, a form of programmed cell death, is independent of cysteine family proteases activation, but relies on the activation of receptor-interacting protein kinase 1 (RIPK1), receptor-interacting protein kinase 3 (RIPK3), and mixed-lineage kinase domain-like (MLKL) [65]. Follicle size and granulosa cells ATP content influence the mode of cell death. While high ATP levels favor apoptosis, low ATP levels induce necrotic morphology in granulosa cells [66,67].

A subtype of acetylcholinesterase (AChE), known as readthrough acetylcholinesterase (AChE-R), induces necroptosis in human ovarian granulosa cells [68]. The oxidative stress associated with PCOS upregulates AchE-R expression, leading to necrotic apoptosis of human granulosa cells and contributing to follicular atresia.

## Pyroptosis of granulosa cells and follicular atresia

Pyroptosis is an inflammatory form of cell death characterized by distinct morphological features including chromatin condensation, cell swelling, and membrane blebbing [65]. This process depends on the activation of specific inflammatory caspases and results in the release of pro-inflammatory factors [69]. Gasdermin D (GSDMD) is a key effector of pyroptosis. Cleavage by caspase-1 generates its N-terminal fragment, which forms pores in the plasma membrane, leading to the release of IL-1β, IL-18, and other cellular contents. Testosterone-treated human granulosa cell lines, mimicking PCOS hyperandrogenism, exhibit pryoptotic characteristics with increased caspase-1 expression, N-terminal fragment of Gasdermin D (N-GSDMD), along with significant upregulation of inflammation-related genes (NLRP3, NF-κB, IL-1β) and proteins (NLRP3, IL-1β, IL-18). This inflammatory response contributes to follicular development and follicular atresia disorders [70]. Xu *et al*. demonstrated that elevated follicular glucose levels in obese patients activate granulosa cell pyroptosis through NLRP3 inflammasomes [71].

## Conclusions

Excessive apoptosis, autophagy, ferroptosis, necroptosis, and pyroptosis of granulosa cells collectively contribute to follicular atresia in PCOS. These distinct yet interconnected cell death pathways can operate independently or synergistically to drive granulosa cell death, ultimately leading to follicular atresia. While the roles of abnormal apoptosis and excessive autophagy in PCOS-induced granulosa cell death and follicular atresia are well-established, recent evidence underscores the importance of ferroptosis and necroptosis in this complex pathophysiological process. Pyroptosis has been infrequently reported in the context of follicular atresia regulation in animal models. PCOS, a complex condition affecting female fertility, has garnered increasing attention in the context of granulosa cell death in follicular atresia, particularly in relation to hyperandrogenism and insulin resistance. A deeper understanding of these processes is crucial for developing effective interventions to improve the endocrine environment and overall well-being of women with PCOS.

## Abbreviations

ACSL4: Acyl-CoA synthetase long-chain family member 4
AMH: Anti-mülerian hormone
ACh: Acetylcholine
AChE: Acetylcholinesterase
AChE-R: Acetylcholin-esterase readthrough
AMPK: Adenosine 5'-mono-phosphate (AMP)-activated protein kinase
ATP: Adenosine triphosphate
BAK: BCL2 antagonist/killer
BPA: bisphenol A
BCL-2: B cell lymphoma-2
BAX: BCL-2 associated X
CD36: Cluster of Differentiation 36
CircRHBG: Rhesus type B glycoprotein
Cyt c: Cytochrome c
CTGF: Connective Tissue Growth Factor
DEHP: di(2-ethylhexyl) phthalate
DHT: dihydrotestosterone
EDCs: endocrine disrupting chemicals
GCs: Granulosa cells
GCLC: Glutamate cysteine ligase which is composed of a catalytic
GSDMD: Gasdermin D
IL-6: Interleukin-6
IL-1β: Interleukin-1beta
IL-18: Interleukin 18
LH: Luteinizing Hormone
mTOR: mammalian target of rapamycin
mTORC1: Mechanistic target of rapamycin form protein complex
N-GSDMD: N-terminal GSDMD
NF-Kb: Nuclear factor-kappa B
NLRP3: NACHT, LRR, and PYD domains-containing protein 3
oxLDL: Oxidized Low-Density Lipoprotein
PCOS: Polycystic ovarian syndrome
PF: Primordial follicles
RNA: Ribonucleic Acid
ROS: Reactive Oxygen Species
ROS: Reactive oxygen species
SHBG: Sex Hormone-Binding Globulin
SLC7A11: Solute carrier family 7 member 11
TFRC: Transferrin receptor
TGF-β: Transforming Growth Factor beta
ULK1: Unc-51-like kinase 1

## References

[1] Ajmal N, Khan SZ, Shaikh R (2019). Polycystic ovary syndrome (PCOS) and genetic predisposition: A review article. Eur J Obstet Gynecol Reprod Biol X.

[2] Zeng X, Xie YJ, Liu YT, Long S-L, Mo Z-C (2020). Polycystic ovarian syndrome: Correlation between hyperandrogenism, insulin resistance and obesity. Clin Chim Acta.

[3] Teede HJ, Misso ML, Costello MF, Dokras A, Laven J, Moran L, Piltonen T (2018). Recommendations from the international evidence-based guideline for the assessment and management of polycystic ovary syndrome. Fertil Steril.

[4] Siddiqui S, Mateen S, Ahmad R, Moin S (2022). A brief insight into the etiology, genetics, and immunology of polycystic ovarian syndrome (PCOS). J Assist Reprod Gene.

[5] Liang A, Huang L, Liu H, He W, Lei X, Li M, Li S (2021). Resveratrol Improves Follicular Development of PCOS Rats by Regulating the Glycolytic Pathway. Mol Nutr Food Res.

[6] Chang HM, Qiao J, Leung PC (2016). Oocyte-somatic cell interactions in the human ovary-novel role of bone morphogenetic proteins and growth differentiation factors. Hum Reprod Update.

[7] Jahromi BN, Mosallanezhad Z, Matloob N, Davari M, Ghobadifar MA (2015). The potential role of granulosa cells in the maturation rate of immature human oocytes and embryo development: A co-culture study. Clin Exp Reprod Med.

[8] Wang X, Yang J, Li H, Mu H, Zeng L, Cai S, Su P (2023). miR-484 mediates oxidative stress-induced ovarian dysfunction and promotes granulosa cell apoptosis via SESN2 downregulation. Redox Biol.

[9] Kulus M, Kranc W, Sujka-Kordowska P, Mozdziak P, Jankowski M, Konverska A, Kulus J (2020). The processes of cellular growth, aging, and programmed cell death are involved in lifespan of ovarian granulosa cells during short-term IVC - Study based on animal model. Theriogenology.

[10] Zhang H, Risal S, Gorre N, Busayavalasa K, Li X, Shen Y, Bosbach B (2014). Somatic cells initiate primordial follicle activation and govern the development of dormant oocytes in mice. Curr Biol.

[11] Stringer JM, Alesi LR, Winship AL, Hutt KJ (2023). Beyond apoptosis: evidence of other regulated cell death pathways in the ovary throughout development and life. Hum Reprod Update.

[12] Ketelut-Carneiro N, Fitzgerald KA (2022). Apoptosis, Pyroptosis, and Necroptosis-Oh My! The Many Ways a Cell Can Die. J Mol Biol.

[13] Liu HL, Meng FX (2019). Effect of oxidative stress on antral follicular atresia in animals and its mechanism. J Nanjing Agricult Univ.

[14] Wang JP, Zhang KY (2021). Effects of Oxidative Stress on Follicular Atresia in Poultry and Its Mechanism. Chin J Animal Nutr.

[15] Zheng K, Yang MG, Yan CJ (2019). Mitochondrial Dynamics and Apoptosis. Chin J Cell Biol.

[16] Rudnicka E, Kunicki M, Calik-Ksepka A, Suchta K, Duszewska A, Smolarczyk K, Smolarczyk R (2021). Anti-Müllerian Hormone in Pathogenesis, Diagnostic and Treatment of PCOS. Int J Mol Sci.

[17] Salehi R, Mazier HL, Nivet A-L, Reunov AA, Lima P, Wang Q, Fiocco A (2020). Ovarian mitochondrial dynamics and cell fate regulation in an androgen-induced rat model of polycystic ovarian syndrome. Sci Rep.

[18] Yang YQ, Wu GX, Yang J (2021). Research progress on the role of heat shock proteins in polycystic ovary syndrome. Prog Obst Gynecol.

[19] Ni XR, Sun ZJ, Hu GH, Wang R-H (2015). High concentration of insulin promotes apoptosis of primary cultured rat ovarian granulosa cells via its increase in extracellular HMGB1. Reprod Sci.

[20] Liu XS, Qiao YY (2021). Research on the progress about the pathogenesis of spontaneous abortion in patients with polycystic ovary syndrome. Chin J Birth Health Hered.

[21] Zhang Q, Ren J, Wang F, Pan M, Cui L, Li M, Qu F (2022). Mitochondrial and glucose metabolic dysfunctions in granulosa cells induce impaired oocytes of polycystic ovary syndrome through Sirtuin 3. Free Radic Biol Med.

[22] Ma Y, Zheng L, Wang Y, Gao Y, Xu Y (2022). Arachidonic Acid in Follicular Fluid of PCOS Induces Oxidative Stress in a Human Ovarian Granulosa Tumor Cell Line (KGN) and Upregulates GDF15 Expression as a Response. Front Endocrinol (Lausanne).

[23] Sun BY, Han SY (2022). Research progress on the relationship between chronic low-grade inflammation and polycystic ovary syndrome. Chin J Birth Health Hered.

[24] Wang D, Weng Y, Zhang Y, Wang R, Wang T, Zhou J, Shen S (2020). Exposure to hyperandrogen drives ovarian dysfunction and fibrosis by activating the NLRP3 inflammasome in mice. Sci Total Environ.

[25] Zhang XL, Li Y, Liu L, Wang Z, Xu F (2016). Effect of Inflammatory Markers on the Expression of P53, Bax, Caspase-3, Bcl-2 and Survivin in Human Granulosa Cells. J Pract Obst Gynecol.

[26] La Merrill MA, Vandenberg LN, Smith MT, Goodson W, Browne P, Patisaul HB, Guyton KZ (2020). Consensus on the key characteristics of endocrine-disrupting chemicals as a basis for hazard identification. Nat Rev Endocrinol.

[27] Akgül S, Sur Ü, Düzçeker Y, Balcı A, Kızılkan MP, Kanbur N, Bozdağ G (2019). Bisphenol A and phthalate levels in adolescents with polycystic ovary syndrome. Gynecol Endocrinol.

[28] Luo Y, Nie Y, Tang L, Xu CC, Xu L (2020). The correlation between UDP-glucuronosyltransferase polymorphisms and environmental endocrine disruptors levels in polycystic ovary syndrome patients. Medicine (Baltimore).

[29] Wang Y, Zhu Q, Dang X, He Y, Li X, Sun Y (2017). Local effect of bisphenol A on the estradiol synthesis of ovarian granulosa cells from PCOS. Gynecol Endocrinol.

[30] Zhan W, Tang W, Shen X, Xu H, Zhang J (2023). Exposure to bisphenol A and its analogs and polycystic ovarian syndrome in women of childbearing age: A multicenter case-control study. Chemosphere.

[31] Al-Saleh I (2022). The relationship between urinary phthalate metabolites and polycystic ovary syndrome in women undergoing in vitro fertilization: Nested case-control study. Chemosphere.

[32] Milankov A, Milanović M, Milošević N, Sudji J, Pejaković S, Milić N, Bjelica A, Stojanoska MM (2023). The effects of phthalate exposure on metabolic parameters in polycystic ovary syndrome. Clin Chim Acta.

[33] Zhan W, Qiu W, Ao Y, Zhou W, Sun Y, Zhao H, Zhang J (2023). Environmental Exposure to Emerging Alternatives of Per- and Polyfluoroalkyl Substances and Polycystic Ovarian Syndrome in Women Diagnosed with Infertility: A Mixture Analysis. Environ Health Perspect.

[34] Gu J, Yuan T, Ni N, Ma Y, Shen Z, Yu X, Shi R (2019). Urinary concentration of personal care products and polycystic ovary syndrome: A case-control study. Environ Res.

[35] Guo Z, Qiu H, Wang L, Wang L, Wang C, Chen M, Zuo Z (2017). Association of serum organochlorine pesticides concentrations with reproductive hormone levels and polycystic ovary syndrome in a Chinese population. Chemosphere.

[36] Prabhu NB, Adiga D, Kabekkodu SP, Bhat SK, Satyamoorthy K, Rai PS (2022). Bisphenol A exposure modulates reproductive and endocrine system, mitochondrial function and cellular senescence in female adult rats: A hallmarks of polycystic ovarian syndrome phenotype. Environ Toxicol Pharmacol.

[37] Yan W, Li M, Guo Q, Li X, Zhou S, Dai J, Zhang J (2022). Chronic exposure to propylparaben at the humanly relevant dose triggers ovarian aging in adult mice. Ecotoxicol Environ Saf.

[38] Liu J, Wang W, Zhu J, Li Y, Luo L, Huang Y, Zhang W (2018). Di(2-ethylhexyl) phthalate (DEHP) influences follicular development in mice between the weaning period and maturity by interfering with ovarian development factors and microRNAs. Environ Toxicol.

[39] Sun J, Gan L, Lv S, Wang T, Dai C, Sun J (2023). Exposure to Di-(2-Ethylhexyl) phthalate drives ovarian dysfunction by inducing granulosa cell pyroptosis via the SLC39A5/NF-κB/NLRP3 axis. Ecotoxicol Environ Saf.

[40] Lin M, Hua R, Ma J, Zhou Y, Li P, Xu X, Yu Z, Quan S (2021). Bisphenol A promotes autophagy in ovarian granulosa cells by inducing AMPK/mTOR/ULK1 signalling pathway. Environ Int.

[41] Du J, Ruan X, Jin F, Li Y, Cheng J, Gu M, Mueck AO (2021). Abnormalities of early folliculogenesis and serum anti-Müllerian hormone in Chinese patients with polycystic ovary syndrome. J Ovarian Res.

[42] Cozzolino M, Herraiz S, Titus S, Roberts L, Romeu M, Peinado I, Scott RT (2022). Transcriptomic landscape of granulosa cells and peripheral blood mononuclear cells in women with PCOS compared to young poor responders and women with normal response. Hum Reprod.

[43] Cheng M, Song Z, Guo Y, Luo X, Li X, Wu X, Gong Y (2023). 1α,25-Dihydroxyvitamin D(3) Improves Follicular Development and Steroid Hormone Biosynthesis by Regulating Vitamin D Receptor in the Layers Model. Curr Issues Mol Biol.

[44] Wan T, Sun H, Mao Z, Zhang L, Chen X, Shi Y, Shang Y (2021). Vitamin D deficiency inhibits microRNA-196b-5p which regulates ovarian granulosa cell hormone synthesis, proliferation, and apoptosis by targeting RDX and LRRC17. Ann Transl Med.

[45] Lejman-Larysz K, Golara A, Baranowska M, Kozłowski M, Guzik P, Szydłowska I, Nawrocka-Rutkowska J (2023). Influence of Vitamin D on the Incidence of Metabolic Syndrome and Hormonal Balance in Patients with Polycystic Ovary Syndrome. Nutrients.

[46] Wu R-X, Dong Y-Y, Yang P-W, Wang L, Deng Y-H, Zhang H-W, Huang X-Y (2019). CD36- and obesity-associated granulosa cells dysfunction. Reprod Fertil Dev.

[47] Chen X, Liu Y, Shan Y, Jin X, Shi Q, Jia C (2017). Oxidized low-density lipoprotein suppresses mouse granulosa cell differentiation through disruption of the hypoxia-inducible factor 1 pathway. Mol Reprod Dev.

[48] Lin X-H, Wang H, Wu D-D, Ullah K, Yu T-T, Rahman TU, Huang H-F (2017). High Leptin Level Attenuates Embryo Development in Overweight/Obese Infertile Women by Inhibiting Proliferation and Promotes Apoptosis in Granule Cell. Horm Metab Res.

[49] Lou Y, Yu W, Han L, Yang S, Wang Y, Ren T, Yu J, Zhao A (2017). ROS activates autophagy in follicular granulosa cells via mTOR pathway to regulate broodiness in goose. Anim Reprod Sci.

[50] Debnath J, Gammoh N, Ryan KM (2023). Autophagy and autophagy-related pathways in cancer. Nat Rev Mol Cell Biol.

[51] Zhou J, Peng X, Mei S (2019). Autophagy in Ovarian Follicular Development and Atresia. Int J Biol Sci.

[52] Xu D, Jiang X, He H, Liu D, Yang L, Chen H, Wu L (2019). SIRT2 functions in aging, autophagy, and apoptosis in post-maturation bovine oocytes. Life Sci.

[53] Escobar ML, Echeverria OM, Palacios-Martínez S, Juárez-Chavero S, Sánchez-Sánchez L, Vázquez-Nin GH (2019). Beclin 1 Interacts With Active Caspase-3 and Bax in Oocytes From Atretic Follicles in the Rat Ovary. J Histochem Cytochem.

[54] Li X, Qi J, Zhu Q, He Y, Wang Y, Lu Y, Wu H, Sun Y (2019). The role of androgen in autophagy of granulosa cells from PCOS. Gynecol Endocrinol.

[55] Xing J, Qiao G, Luo X, Liu S, Chen S, Ye G, Zhang C, Yi J (2023). Ferredoxin 1 regulates granulosa cell apoptosis and autophagy in polycystic ovary syndrome. Clin Sci (Lond).

[56] Stockwell BR (2022). Ferroptosis turns 10: Emerging mechanisms, physiological functions, and therapeutic applications. Cell.

[57] Martínez-García MA, Luque-Ramírez M, San-Millán JL, Escobar-Morreale HF (2009). Body iron stores and glucose intolerance in premenopausal women: role of hyperandrogenism, insulin resistance, and genomic variants related to inflammation, oxidative stress, and iron metabolism. Diabetes Care.

[58] Escobar-Morreale HF, Luque-Ramírez M, Alvarez-Blasco F, Botella-Carretero, Sancho J, San Millán JL (2005). Body iron stores are increased in overweight and obese women with polycystic ovary syndrome. Diabetes Care.

[59] Escobar-Morreale HF Luque-Ramírez M Role of androgen-mediated enhancement of erythropoiesis in the increased body iron stores of patients with polycystic ovary syndrome Fertil Steril 2011 95 1730 1735e1. 10.1016/j.fertnstert.2011.01.038 21300335

[60] Escobar-Morreale HF, San Millán JL (2007). Abdominal adiposity and the polycystic ovary syndrome. Trends Endocrinol Metab.

[61] Zhang L, Wang F, Li D, Yan Y, Wang H (2021). Transferrin receptor-mediated reactive oxygen species promotes ferroptosis of KGN cells via regulating NADPH oxidase 1/PTEN induced kinase 1/acyl-CoA synthetase long chain family member 4 signaling. Bioengineered.

[62] Huang X, Geng H, Liang C, Xiong X, Du X, Zhuan Q, Liu Z (2025). Leonurine restrains granulosa cell ferroptosis through SLC7A11/GPX4 axis to promote the treatment of polycystic ovary syndrome. Free Radic Biol Med.

[63] Zhang Y, Hu M, Jia W, Liu G, Zhang J, Wang B, Li J (2020). Hyperandrogenism and insulin resistance modulate gravid uterine and placental ferroptosis in PCOS-like rats. J Endocrinol.

[64] Zhang D, Yi S, Cai B, Wang Z, Chen M, Zheng Z, Zhou C (2021). Involvement of ferroptosis in the granulosa cells proliferation of PCOS through the circRHBG/miR-515/SLC7A11 axis. Ann Transl Med.

[65] Snyder AG, Oberst A (2021). The Antisocial Network: Cross Talk Between Cell Death Programs in Host Defense. Annu Rev Immunol.

[66] Ying Y, Padanilam BJ (2016). Regulation of necrotic cell death: p53, PARP1 and cyclophilin D-overlapping pathways of regulated necrosis?. Cell Mol Life Sci.

[67] Wakizoe E, Ashizawa K, Sakamoto SH, Hemmi K, Kobayashi I, Tsuzuki Y (2015). The effects of extracellular ATP and its receptor antagonists on pig oocytes during in vitro maturation. Zygote.

[68] Blohberger J, Kunz L, Einwang D, Berg U, Berg D, Ojeda SR, Dissen GA (2015). Readthrough acetylcholinesterase (AChE-R) and regulated necrosis: pharmacological targets for the regulation of ovarian functions?. Cell Death Dis.

[69] Ding J, Wang K, Liu W, She Y, Sun Q, Shi J, Sun H (2016). Pore-forming activity and structural autoinhibition of the gasdermin family. Nature.

[70] Xiang Y, Ding HM, Chen T, Xu TY, Ge HS (2023). Hyperandrogenism affects ovarian granulosa cell proliferation by inducing pyroptosis. J Repr Med.

[71] Xu R, Zhao H, Qi J, Yao G, He Y, Lu Y, Zhu Q (2023). Local glucose elevation activates pyroptosis via NLRP3 inflammasome in ovarian granulosa cells of overweight patients. FASEB J.

